# The Relationship between Ischemic Optic Neuropathy and Internal Carotid Artery Dissection: A Systematic Review

**DOI:** 10.3390/jcm13092486

**Published:** 2024-04-24

**Authors:** Matteo Ripa, Neeraj Apoorva Shah, Chiara Schipa, Paola Aceto, Tommaso Donati

**Affiliations:** 1 Department of Ophthalmology, Sankara Eye Hospital, Jaipur 302039, Rajasthan, India; 2 Catholic University “Sacro Cuore”, 00168 Rome, Italy; 3 Department of Emergency, Anesthesiological and Reanimation Sciences, Fondazione Policlinico Universitario A. Gemelli IRCCS, 00168 Rome, Italy; 4 Unit of Vascular Surgery, Fondazione Policlinico Universitario A. Gemelli IRCCS, 00168 Rome, Italy

**Keywords:** internal carotid artery dissection, non-arteritic ischemic optic neuropathy, posterior ischemic optic neuropathy

## Abstract

**Background**: To evaluate and review the current evidence regarding the association between ischemic optic neuropathy (ION) and internal carotid artery dissection (ICAD). **Methods**: We systematically reviewed studies according to the Preferred Reporting Items for Systematic Reviews and Meta-Analysis guidelines (PRISMA), searching three databases (Scopus, Pubmed, and Embase) for relevant articles that clearly described the correlation between ION and ICAD. All studies that examined the association between ICAD and the development of ION were synthesized. Quality assessment using the Newcastle–Ottawa Scale (NOS) and Joanna Briggs Institute (JBI) Critical Appraisal Checklist for Case Reports and Case Series were conducted. **Results**: Our search yielded 198 manuscripts published in the English language. Following study screening, fourteen studies were selected. The number of participants with ION following ICAD ranged from one to four, with sixteen patients experiencing either anterior ION, posterior ION, or a combination of both. The anterior or posterior ischemic optic neuropathy (AION and PION) patients’ ages were 48.75 ± 11.75 and 49.62 ± 12.85, respectively. Fourteen out of sixteen patients experienced spontaneous ICAD, whereas the traumatic etiology was ascertained in two patients. **Conclusions**: Hence, albeit rare, ophthalmologists should consider ICAD a potential cause of ION, especially in young adults with concomitant cephalic pain and vision reduction.

## 1. Introduction

Internal carotid artery dissection (ICAD) represents one of the most important causes of ischemic cerebrovascular symptoms in younger patients worldwide, accounting for 2.5% of all stroke cases and for up to 5% to 22% of those in patients under 45 years of age [1,2]. It is classified according to the type of artery involved (vertebral or carotid) and the location of the dissected artery (intracranial or extracranial). The extracranial internal carotid artery (ICA) dissection represents the most prevalent form [3].

During carotid artery dissection (CAD), arterial blood penetrates the vascular wall via the injured carotid intima, separating the intimal and medial layers. This procedure causes the creation of an intramural hematoma, which results in stenosis or occlusion. As a result, CAD can produce thrombosis and vascular stenosis, leading to an ischemic stroke or transient ischemic attack [4].

Despite being often accompanied by local symptoms, such as head, facial, or neck pain and tinnitus, several ophthalmic manifestations related to ICAD have been widely reported [5]. To be specific, postganglionic oculosympathetic paralysis [6], transient monocular blindness, permanent visual loss [5], homonymous visual field defect [7], ocular ischemic syndrome [8], cranial nerve palsy [9], and transient cilioretinal artery occlusion [10] have been previously reported.

In addition, it has been reported that patients with carotid artery stenosis and occlusion may occasionally experience either anterior or posterior ischemic optic neuropathy (AION and PION), which represent a spectrum of several different types characterized by the infarction of the optic nerve, caused by compromised circulation in the tiny arteries supplying its anterior or posterior part [11].

ION is classified into two types, based on the location of the ischemic damage: AION, which involves the anterior part of the optic nerve, supplied by the posterior ciliary artery circulation, and PION, which involves the remaining part of the optic nerve, supplied by the pial capillary plexus [11].

Ischemic optic neuropathy (ION) is clinically characterized by sudden, painless, monocular vision loss associated with relative pupillary afferent and classically altitudinal visual field defects, and the AION represents the most frequent cause of acute optic neuropathy in patients over 50 years of age, with an estimated prevalence of 2.3 to 10.2 per 100,000 [12,13].

The fundoscopy exam in AION reveals hyperemic optic disc swelling, while in PION, it remains normal. Regardless of the location of the ischemic damage, the optic disc turns pale about a month after visual impairment [14].

The ICA is responsible for the optic nerve blood supply, as the central retinal artery and posterior ciliary artery supply its anterior portion, whereas the multiple pial vessels arising from the ophthalmic artery supply the posterior portion [13,15]. Therefore, the ICA’s occlusion leads to the reduced blood supply of the optic nerve, determining AION and PION [16]. Nonetheless, AION and PION have been rarely reported as either initial or accompanying symptoms of ICAD [16,17,18,19,20,21,22,23,24,25,26,27,28,29].

To the best of our knowledge, no systematic reviews have investigated the role of ICAD in the onset of ION. Therefore, our research aimed to systematically review the current evidence and critically evaluate the association between ION and ICAD.

## 2. Materials and Methods

### 2.1. Search Strategy

To evaluate the association between ION and ICAD, a systematic review was conducted according to the Preferred Reporting for Systematic Reviews and Meta-Analyses (PRISMA) guidelines [30]. The research was registered in the International Prospective Register of Systematic Reviews (PROSPERO) database (identifier: CRD42024519388).

Three databases (Embase, Scopus, and PubMed) were reviewed until 21 February 2024, using free text to evaluate the association between ION and ICAD. The search strategy combined the keywords based on recommendations from every database. Specifically, the Medical Subject Headings (MeSH) were adopted to find articles in PubMed, whereas the Embase Subject Headings were utilized in the EMBASE. The keywords were chosen according to related subject studies and used with Boolean operators, such as OR and AND, to extend, restrict, and direct the search. Furthermore, the whole search process was conducted without database filters to avoid hypothetical loss. Our key search consisted of the following terms: “Carotid” AND “dissection”, AND “optic neuropathy”, OR “ION”, OR “PION”, OR “AION”. This process continued until we attained a stage where adding more terms produced no new results. In addition, the included articles were also hand-searched to identify further studies that could have potentially been missed during the initial database search. Supplementary Materials S1 and S2 report the detailed search strategy and PRISMA checklist.

### 2.2. Eligibility Criteria

To evaluate the association between ION and ICAD, we included all available randomized and nonrandomized clinical trials, case reports, retrospective and prospective case series, and cross-sectional, cohort, and case-control studies, clearly assessing the association between the above-mentioned conditions. 

ION was defined as a sudden visual loss in one eye, accompanied by relative afferent pupillary defect; normal inflammatory markers, such as erythrocyte sedimentation rate or C-reactive protein; visual field defect on the perimetry; and an absence of any other ocular, optic nerve, or neurologic disorders, including compressive, inflammatory, or other mechanisms that might influence or explain the patient’s visual symptoms. Specifically, AION was considered if the initial fundus showed disc edema and/or an early filling defect of the disc by fluorescein angiography (FFA) in the affected eye. In contrast, PION was ascertained if the optic disc and the rest of the fundus on the ophthalmoscopy and FFA were normal with the development of a diffuse or segmental optic disc pallor after a few weeks. Therefore, only papers including patients diagnosed with non-arteritic anterior and non-arteritic posterior optic neuropathy associated with ICAD were included.

ICAD (spontaneous or traumatic) was defined as stenosis in the ICA, due to an intramural hematoma, resulting in carotid artery stenosis or occlusion. 

We exempted the following studies from our analysis: (1) conference abstracts, (2) narrative reviews, (3) theses, (4) book sections, and (5) non-English-language studies. In addition, studies reporting incomplete data were also excluded. Finally, studies including patients with concomitant arteritic ischemic optic neuropathies and ICAD or ION associated with any other ophthalmic disease affecting the optic disc blood supply or with any neurologic disease affecting the optic nerve, such as multiple sclerosis, Alzheimer’s, and Parkinson’s disease, were further excluded.

### 2.3. Study Selection, Data Collection, and Risk of Bias Assessment

To identify articles reporting an association between ION and ICAD, two authors (M.R. and T.D.) independently screened the titles and abstracts of the retrieved studies, as well as the full text of the remaining studies. Studies whose designs failed to fulfill the inclusion criteria were not included. Moreover, the baseline and outcomes data were further independently extracted. If a consensus could not be achieved, the investigators discussed the discrepancies for adjudication with a third author (P.A.). Reasons for exclusion were recorded. Whenever the article could not be retrieved, or additional information was required, corresponding authors of eligible studies were contacted. Consequently, the data were gathered directly from the included research or supplied by the corresponding authors. We extracted the following data from each article: first author, year published, country, study design, total sample size, number of patients with ION, number of patients in control groups (if available), mean patients’ age, gender, involved side, type of ION, study time, type of dissection (traumatic vs. spontaneous) and occlusion site (if available), mean time between ION onset and first consultation (if available), follow-up duration (if available), diagnostic investigation used for diagnosis, and stenosis severity. The Covidence systematic review software© (Veritas Health Innovation, Melbourne, Australia), available at www.covidence.org [31] (accessed on 1 March 2024), was employed to record and evaluate the study data until 1 March 2024. 

Two authors (M.R. and T.D.) independently appraised each study’s methodological quality, comparing the quality assessment data and discussing the discrepancies for adjudication in case a consensus could not be achieved with a third author (P.A.). The Newcastle–Ottawa Scale (NOS) [32] was employed for the cross-sectional studies, and the Joanna Briggs Institute (JBI) Critical Appraisal Checklist for Case Reports and the JBI Critical Appraisal Checklist for Case Series were employed for the quality assessment of the case reports and case series, respectively [33]. 

## 3. Results

### 3.1. Study Selection

The search provided 198 indexed articles (56, 60, and 82 records from Scopus, Embase, and PubMed, respectively). No further articles were retrieved after the reference list’s search. A total of 123 articles were screened after the duplication removal. A total of 103 studies were excluded after the title and abstract screenings, and only 19 full-text articles were retrieved, evaluated, and deemed entirely eligible. In addition, five articles were omitted due to irrelevant outcomes or inappropriate study design. 

**Figure 1:**
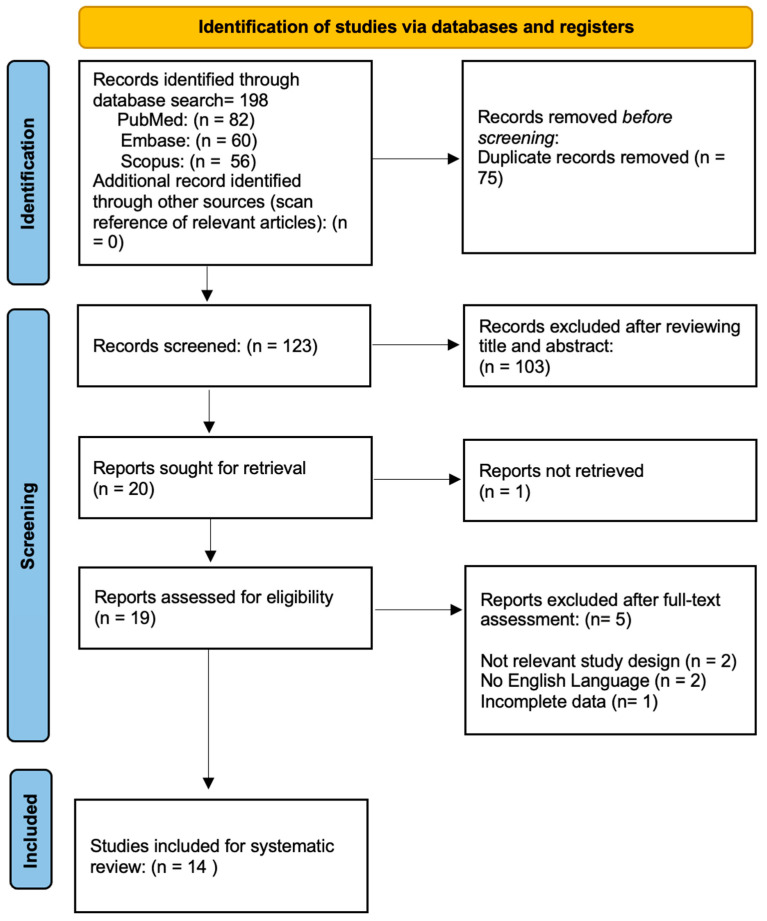
Flow diagram of the study selection process.

Finally, as per inclusion and exclusion criteria, 14 articles were included in the systematic review [16,17,18,19,20,21,22,23,24,25,26,27,28,29] (Figure 1).

### 3.2. Study Characteristics

Table S1, available in Supplementary Material S3, summarizes the main characteristics of the first author, year published, country, study design, total sample size, number of patients with ION, number of patients in control groups (if available), mean patients’ age, gender, involved side, type of ION, study time, type of dissection (traumatic vs. spontaneous) and occlusion site (if available), mean time between ION onset and first consultation (if available), follow-up duration (if available), diagnostic investigation used for diagnosis, and stenosis severity.

The included articles were published between 2 April 1990 [16] and 1 December 2021 [22].

Two cross-sectional studies [23,27], eleven case reports [16,17,18,19,20,21,22,24,25,28,29], and one case series [26] were analyzed. The number of participants with ION ranged from 1 [16,17,18,19,20,21,22,24,25,28,29] to 4 [26], with a total of 16 patients experiencing either AION (5 out 16), PION (10 out 16), or a combination of both (1 out 16). Eleven articles reported the ION patients’ age [(median ± standard deviation), (margin of error)]: 48.35 ±11.83 (±6.54%) [17,18,19,20,21,22,24,25,26,28,29].

**Figure 2:**
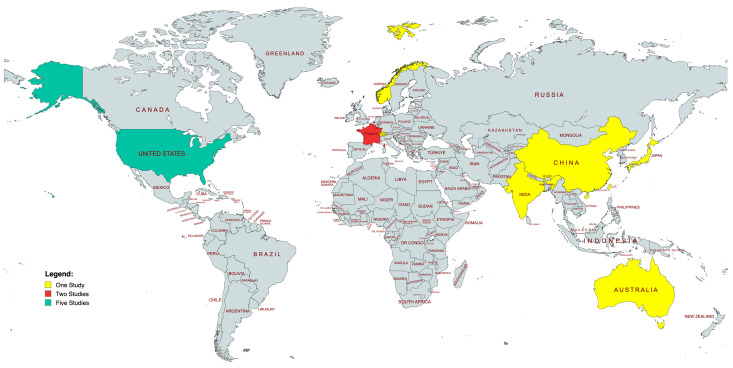
World map of studies included. Map generated through MapChart (MapChart, 2021).

To be specific, the AION patients’ age [(median ± standard deviation), (margin of error)] was 48.75 ± 11.75 (±12.06%) [18,22,25,26], whereas the PION patients’ age [(median ± standard deviation), (margin of error)] was 49.62 ± 12.85 (±9.16%) [16,17,20,21,24,25,26,28,29]. Overall, four studies involved patients from European countries [21,23,26,27], five involved patients from American countries [16,17,18,22,29], four involved patients from Asian countries [19,20,24,25], and one study involved patients from Australia [28] (Figure 2).

Fourteen out of sixteen patients experienced spontaneous ICAD [16,17,19,20,21,22,23,24,26,27,28], whereas the traumatic etiology was ascertained in two patients [18,29]. Stenosis severity was reported in ten studies [18,19,21,22,24,25,26,27,28,29], with the majority reporting it as a range (0–100% or mild–moderate and severe) [18,19,21,22,24,25,26,27,28,29].

Regarding the treatment of ICAD, most papers mentioned using antithrombotic therapies, such as antiplatelet and anticoagulation agents, to prevent subsequent emboli from dissection and to decrease the risk of subsequent ischemic stroke. Three out of fourteen studies did not mention the adopted treatment [16,17,28]. In contrast, Anders et al. reported that the anticoagulant agents were not administered due to the subdural hematoma, which was due to the acute trauma [29]. Beyond the medical therapy, further treatments, such as recanalization of the dissected carotid artery, were reported by 2 out of 16 papers [19,23].

### 3.3. Risk of Bias

All studies’ risks of bias evaluation are summarized in Tables S1–S3, available in Supplementary Material S4. Most of the studies scored one or two in the quality scale’s significant domains. The quality rating of the cross-sectional studies averaged seven (95% CI 5.6 to 8.4) out of the maximum score on the NOS [23,27]. According to the JBI Critical Appraisal Checklist for Case Series, the quality of the included studies ranged from low to good. Most case reports gained four out of eight quality criteria or more [16,17,18,19,20,21,22,24,25,28,29]. The case series scored nine out of ten quality criteria. Indeed, it extensively provided information regarding population demographics [26].

## 4. Discussion

To the best of our knowledge, this is the first systematic review analyzing the association between ION and ICAD.

A range of ocular symptoms, including Horner’s syndrome, amaurosis fugax, hemianopia, sixth cranial nerve palsy, and ischemic optic neuropathy, may occur in patients with ICA dissection. In these patients, ION may even represent its initial ocular manifestation [25]. Nonetheless, less than 20 cases of ION have been reported in patients with carotid artery stenosis and/or occlusion, either spontaneous or traumatic, related to carotid artery dissection [16,17,18,19,20,21,22,23,24,25,26,27,28,29]. Indeed, the estimated non-arteritic AION secondary to ICAD prevalence is 1.8% among patients with ICAD. Specifically, Biousse et al. reported that optic neuropathy occurred in only 2.5% of patients (4 out of 166) who had carotid artery dissection [26]. In 1980, McNeil et al. reported a case of left extracranial ICAD associated with “an indistinct temporal disc margin on fundoscopy” and blurry vision exacerbated by changes in posture [34], and afterward, Rivkin et al. described a man who presented with a painful PION caused by ICAD beginning 2 cm above the bifurcation, followed two days later by a massive cerebral infarct [16].

As previously postulated by Biousse et al., ICAD is thought to cause ION by reducing ocular blood flow due to a sudden reduction in the true lumen’s caliber, which can lower the perfusion pressure in the optic nerve head and the ocular vascular bed, causing progressive nerve damage and ultimately increasing the risk of developing ION.

To be specific, in 1998, the authors described four cases of ION in 110 consecutive patients with CAD. They found that these patients had neither central retinal artery occlusion, ischemic ocular syndrome, nor cerebral infarction. They found that the preserved supply through choroidal anastomoses during the early phase and rapid re-canalization of the ICA in all of their patients could have explained the relative retinal sparing [26].

Traumatic ICAD associated with IONs was rarely reported. ICAD following blunt trauma could be caused by different mechanisms, such as a direct application of force to the neck, a hyperextension and contralateral rotation of the head and neck, an intraoral trauma affecting the carotid artery at the angle of the jaw, or lacerations of the carotid artery resulting from a basilar skull fracture [35]. Local or neurological symptoms typically begin within 72 h of injury, although there are reports of delayed symptoms for up to six months [36]. As a result, CAD caused by blunt trauma injuries may not be detected promptly; indeed, some patients may not even show symptoms until neurological symptoms manifest. In 2000, Babovic et al. reported a high-speed motor vehicle accident that led to ICAD, followed by anterior ION five days after surgery for the reduction and internal fixation of the facial fractures [18]. On the fifth postoperative day, the patient reported decreased visual acuity in her right eye in the early morning, which progressed to no light perception. Afterward, the magnetic resonance imaging (MRI) revealed that the carotid arteries were dissected bilaterally, involving the intracranial portion of both internal carotid arteries, with 50% stenosis on the right side. Despite the anticoagulant therapy, no further improvement in visual acuity was detected, and the patient developed optic disc atrophy.

In 2016, Anders et al. reported the first case of traumatic superior orbital fissure syndrome in a 35-year-old man who sustained blunt trauma from being struck by a golf club, causing orbital fractures, a right superior orbital fissure fracture, a retained metallic foreign body in the right sphenoid sinus, and a right frontoparietal subdural hematoma, with loss of vision from PION due to the right ICAD caused by the osseous erosion of the posterolateral wall of the sphenoid sinus. They postulated that the previously weakened lateral wall of the sphenoid sinus and the following fracture ruptured the posterolateral wall of the right sphenoid sinus, causing intracranial hemorrhage and the dissection of the right ICA, inducing a secondary PION [29].

Regardless of the underlying cause, the clinical features of ION in patients with ICAD diverge from those of “classic” NA-ION patients. Indeed, the patient’s mean age was actually relatively low—44 years (range: 33–51 years) compared to 60 years (range: 11–90 years). Furthermore, the predominant symptoms were ipsilateral ocular pain and acute cephalic pain. Therefore, ICAD should be taken into consideration when pain, recurrent transient monocular blindness, and Horner syndrome are present in young patients with ION and concomitant severe cephalic pain.

Regarding the treatment of patients experiencing concomitant acute ICAD and ION, in the absence of data from randomized controlled trials, the management of CAD and its complications is often empirical. In the acute phase, the risk of an ischemic stroke that might occur up to one month after the initial dissection symptoms can be reduced by using antiplatelets and anticoagulant medications, such as warfarin and heparin [37]. In addition, Biousse et al. reported that they stopped the antihypertensive agents to improve the brain and eyes’ perfusion, considering the ION-related decreased perfusion pressure [26]. Nowadays, endovascular therapy involving stenting is advised to treat secondary vascular complications, such as aneurysms and severe stenosis, or in cases where medical therapy has failed [38,39]. Furthermore, patients with neurologic symptoms like headache episodes, thromboembolic events, or Horner syndrome should also consider endovascular therapy [40,41].

To the best of our knowledge, this is the first systematic review analyzing the association between ICAD and ION. One of its primary merits is that our results are broadly applicable, as the included studies collected data from patients across various nations over an extended period of time (40 years). Despite representing a strength, on the other hand, including data from patients from different eras, which means different diagnostic tools and clinical understanding, could have biased our results. Therefore, this systematic review has several limitations. Indeed, it involves data mostly retrieved from case reports and case series, whose uncontrolled study designs are known for their increased risk of bias. In addition, regardless of the study design, most of the included papers report data retrieved from tiny sample sizes. Therefore, research with different study designs, such as longitudinal or cross-sectional studies with bigger samples, is required to further investigate the role of ICAD in the onset of IONs. Moreover, no statistical analysis was performed, due to the lack of data amenable to meta-analysis. Finally, we only aimed to evaluate the role of ICAD in ION development without reporting the other varied ocular manifestations of ICAD that could help and, at the same time, may interfere with diagnosis.

## 5. Conclusions

Despite many ophthalmologic signs and symptoms promptly pointing to an ICAD, IONs should be considered a possible ocular feature in ICADs. Hence, albeit rare, ophthalmologists should consider ICAD a potential cause of ION, especially in young adults with concomitant cephalic pain and vision reduction. Accordingly, a close collaboration between clinicians of different subspecialties may lead to prompt diagnosis and treatment that could allow the avoidance of sight- and life-threatening complications, such as retinal ischemia and stroke.

## Data Availability

Not applicable.

## Supplementary Materials

The following supporting information can be downloaded at: https://www.mdpi.com/article/10.3390/jcm13092486/s1, Supplementary Material S1: Detailed research strategy. Supplementary Material S2: PRISMA checklist; Supplementary Material S3: Study characteristics; Supplementary Material S4: Risk of bias evaluation.
