# Supplementary material for: The Relationship between Ischemic Optic Neuropathy and Internal Carotid Artery Dissection: A Systematic Review
Source: J Clin Med. 2024 Apr 24;13(9):2486. doi: 10.3390/jcm13092486 (PMC11084818; doi:10.3390/jcm13092486)
Supplement: Supplementary file 1 [file jcm-13-02486-s001.zip › Supplementary material pdf/Supplementary Material S4.pdf]

Table S1: Newcastle–Ottawa Scale for Critical Appraisal of Cross-Sectional Studies

		Selection				Comparability	Outcome		
**Source**	**Study design**	**Representativeness of the sample**	**Sample size**	**Non-respondents**	**Ascertainment of exposure**	**Based on design and analysis**	**Assessment of outcome**	**Statistical test**	**Total score**
Biousee et al., 1998	Cross-sectional	+1 (b)	+1 (a)	0 (c)	+2 (a)	+2 (a)	+2 (b)	0 (b)	8/10
Kerty, 1998	Cross-sectional	+1 (b)	+1 (a)	0 (c)	+2 (a)	0 (b)	+2 (b)	0 (b)	6/10

Representativeness of the sample: a) Truly representative of the average in the target population. b) Somewhat representative of the average in the target population; c) Selected group of users d) No description of the sampling strategy; Sample size: a) Justified and satisfactory. b) Not justified; Non-respondents: a) Comparability between respondents and non-respondents characteristics is established, and the response rate is satisfactory. b) The response rate is unsatisfactory, or the comparability between respondents and non-respondents is unsatisfactory. c) No description of the response rate or the characteristics of the responders and the non-responders; Ascertainment of the exposure: a) Validated measurement tool. b) Non-validated measurement tool, but the tool is available or described. c) No description of the measurement tool; Comparability: 1) The subjects in different outcome groups are comparable, based on the study design or analysis. Confounding factors are controlled. a) The study controls for the most important factor (select one). b) The study control for any additional factor; Outcome: 1) Assessment of the outcome: a) Independent blind assessment; b) Record linkage; c) Self report; d) No description. 2) Statistical test: a) The statistical test used to analyze the data is clearly described and appropriate, and the measurement of the association is presented, including confidence intervals and the probability level (p value); b) The statistical test is not appropriate, not described or incomplete.

Table S2: Quality assessment of the included case series using the Joanna Briggs Institute (JBI) Critical Appraisal Checklist for Case Series

Source	Were there clear criteria for inclusion in the case series?	Was the condition measured in a standard, reliable way for all participants included in the case series?	Were valid methods used for identification of the condition for all participants included in the case series?	Did the case series have consecutive inclusion of participants?	Did the case series have complete inclusion of participants?	Was there clear reporting of the demographics of the participants in the study?	Was there clear reporting of clinical information of the participants?	Were the outcomes or follow up results of cases clearly reported?	Was there clear reporting of the presenting site(s)/clinic(s) demographic information?	Was statistical analysis appropriate?
Biousee et al., 1998	yes	yes	yes	yes	yes	yes	yes	yes	yes	no

Table S3: Joanna Briggs Institute Critical Appraisal Checklist for Case Reports

Source	Were the criteria for inclusion in the sample clearly defined?	Were the study subjects and setting described in detail?	Was the exposure measured in a valid and reliable way?	Were objective, standard criteria used for measurement of the condition?	Were confounding factors identified?	Were strategies to deal with confounding factors stated?	Were the outcomes measured in a valid and reliable way?	Was appropriate statistical analysis used?
Rivkin et al., 1990	no	yes	yes	yes	no	no	yes	Not applicable
Tsai et al., 1997	yes	yes	yes	yes	no	no	yes	Not applicable
Archer et al., 1998	yes	yes	yes	yes	no	no	yes	Not applicable
Babovic et al., 2005	yes	yes	yes	yes	yes	yes	yes	Not applicable
Koch et al., 2005	yes	no	no	yes	yes	no	no	Not applicable
Kawabe et al., 2009	yes	yes	yes	yes	yes	yes	yes	Not applicable
Lysandropoulos et al., 2010	yes	yes	yes	yes	yes	yes	yes	Not applicable
Anders et al. 2014	yes	yes	no	yes	no	no	yes	Not applicable
Jah et al., 2019	yes	yes	yes	yes	no	no	yes	Not applicable
Zheng et al., 2020	yes	yes	yes	yes	yes	yes	yes	Not applicable
Lains et al., 2021	yes	yes	yes	yes	no	no	yes	Not applicable
