# Supplementary material for: The Relationship between Ischemic Optic Neuropathy and Internal Carotid Artery Dissection: A Systematic Review
Source: J Clin Med. 2024 Apr 24;13(9):2486. doi: 10.3390/jcm13092486 (PMC11084818; doi:10.3390/jcm13092486)
Supplement: Supplementary file 1 [file jcm-13-02486-s001.zip › Supplementary material pdf/Supplementary Material S1.pdf]

Supplementary Material S1: Detailed Search Strategy

PUBMED:
Free Text and MESH

Query: ("optic neuropathy, ischemic"[MeSH Terms] OR ("optic"[All Fields] AND "neuropathy"[All Fields] AND "ischemic"[All Fields]) OR "ischemic optic neuropathy"[All Fields] OR "naion"[All Fields] OR ("ions"[Supplementary Concept] OR "ions"[All Fields] OR "ion"[All Fields] OR "ions"[MeSH Terms]) OR ("mesons"[MeSH Terms] OR "mesons"[All Fields] OR "pion"[All Fields]) OR ("ions"[Supplementary Concept] OR "ions"[All Fields] OR "ion"[All Fields] OR "ions"[MeSH Terms]) OR ("optic nerve diseases"[MeSH Terms] OR ("optic"[All Fields] AND "nerve"[All Fields] AND "diseases"[All Fields]) OR "optic nerve diseases"[All Fields] OR ("optic"[All Fields] AND "neuropathy"[All Fields]) OR "optic neuropathy"[All Fields])) AND ("carotid arteries"[MeSH Terms] OR ("carotid"[All Fields] AND "arteries"[All Fields]) OR "carotid arteries"[All Fields] OR "carotid"[All Fields] OR "carotids"[All Fields] OR "carotidal"[All Fields] OR "carotide"[All Fields] OR "carotideal"[All Fields]) AND ("dissect"[All Fields] OR "dissected"[All Fields] OR "dissecting"[All Fields] OR "dissection"[MeSH Terms] OR "dissection"[All Fields] OR "dissections"[All Fields] OR "dissects"[All Fields])

#	**Search Term**	**No. Results**
1	"Carotid"	157.975
2	"optic neuropathy" OR "NAION" OR "ION" OR "PION" OR "AION"	1.610.291
3	"dissection"	233.878
4	1 AND 2 AND 3	82

EMBASE
Free Text

Mapping options enabled:
- map to preferred term in Emtree
- search also as free text in all fields
- explode using narrower Emtree terms
- search as broadly as possible

#	Search Term	No. of Results
1	(("Carotid" AND "optic neuropathy" OR "AION" OR "ION" OR "PION" AND "dissection")	60

SCOPUS
Free Text

#	Search Term	No. of Results
1	(TITLE-ABS-KEY (carotid) AND TITLE-ABS-KEY (optic AND neuropathy) OR OR TITLE-ABS-KEY (ion) OR TITLE-ABS-KEY (pion) OR TITLE-ABS-KEY (ion) AND TITLE-ABS-KEY (dissection))	56
