# Supplementary material for: The Relationship between Ischemic Optic Neuropathy and Internal Carotid Artery Dissection: A Systematic Review
Source: J Clin Med. 2024 Apr 24;13(9):2486. doi: 10.3390/jcm13092486 (PMC11084818; doi:10.3390/jcm13092486)
Supplement: Supplementary file 1 [file jcm-13-02486-s001.zip › Supplementary material pdf/Supplementary Material S3.pdf]

Table S1: Characteristics of studies included in the systematic review

First Author	Country	Study Design	Total Sample Size (n.)<br><br>Number of Patients with ION (n. and %)<br><br>Number of Patients of control groups (n. and %)	Age (years, mean ± SD or mean and range or mean age and 95% CI)<br><br>Gender (n. and %)<br><br>Involved Side	Study Time	Type of ION	Type of Dissection and occlusion site	Mean time between ION onset and first consultation	Follow-up Duration	Diagnostic investigation used for diagnosis	Stenosis severity
Rivkin et al., 1990	USA	Case Report	1<br><br>1 (100%)<br><br>N/a	N/a<br><br>Female<br><br>Right	N/a	PION	Spontaneous Dissection<br><br>2 cm above the carotid bifurcation	24 hours	N/a	Angiography	N/a
Tsai et al., 1997	Taiwan	Case-Report	1<br><br>1 (100%)<br><br>N/a	55<br><br>Male<br><br>Right	N/a	PION	Spontaneous Dissection<br><br>3 cm above the bifurcation	3 days	4 weeks	Angiography	N/a

First Author	Country	Study Design	Total Sample Size (n.)<br><br>Number of Patients with ION (n. and %)<br><br>Number of Patients of control groups (n. and %)	Age (years, mean ± SD or mean and range or mean age and 95% CI)<br><br>Gender (n. and %)<br><br>Involved Side	Study Time	Type of ION	Type of Dissection and occlusion site	Mean time between ION onset and first consultation	Follow-up Duration	Diagnostic investigation used for diagnosis	Stenosis severity
Biousee et al., 1998	France	Case Series	Total: 110<br><br>AION: 2 (1.8%)<br><br>PION: 2 (1.8%)	Patient 1: 33<br>Patient 2: 41<br>Patient 3: 51<br>Patient 4: 51<br><br>Patient 1: female<br>Patient 2: male<br>Patient 3: female<br>Patient 4: female<br><br>Patient 1: right<br>Patient 2: right<br>Patient 3: left<br>Patient 4: right	14 years	AION<br>PION	Patient 1: Spontaneous Dissection<br><br>Infrapetrous portion<br><br>Patient 2: Spontaneous Dissection<br><br>Cervical portion of the right ICA extending up to its supraclinoid segment<br><br>Patient 3: Spontaneous Dissection<br><br>Cervical segment extending into the petrous portion of the left ICA<br><br>Patient 4: Spontaneous Dissection<br><br>Infrapetrous portion	N/a	N/a	Total Sample: 72 patients: Angiography<br><br>38: MRA<br><br>Patient 1: Angiography<br>Patient 2: Angiography<br>Patient 3: Angiography<br>Patient 4: Angiography	Patient 1: 95%<br>Patient 2: 80%<br>Patient 3: 95%<br>Patient 4: 90%
Biousee et al., 1998	France	Cross-Sectional	Total: 146<br><br>AION: 2 (1.25%)<br>PION: 2 (1.25%)<br><br>N/a	Total: 45.5 (14 to 71)<br><br>Female: 67 (45.9%)<br>Male: 79 (54.1%)<br><br>N/a	25 years	AION<br>PION	Traumatic and spontaneous Dissections<br><br>N/a	N/a	N/a	Total Sample: 77 patients: Angiography<br><br>69: MRA	80-95%

First Author	Country	Study Design	Total Sample Size (n.)<br><br>Number of Patients with ION (n. and %)<br><br>Number of Patients of control groups (n. and %)	Age (years, mean ± SD or mean and range or mean age and 95% CI)<br><br>Gender (n. and %)<br><br>Involved Side	Study Time	Type of ION	Type of Dissection and occlusion site	Mean time between ION onset and first consultation	Follow-up Duration	Diagnostic investigation used for diagnosis	Stenosis severity
Archer et al., 1998	Australia	Case Report	1<br><br>1 (100%)<br><br>N/a	52<br><br>Man<br><br>Left	N/a	PION	Spontaneous Dissection<br><br>2 cm below the skull base	N/a	10 months	CTA and MRA	100%
Kerty, 1998	Norway	Cross-Sectional	Total: 28<br><br>PION: 1 (3.5%)<br><br>N/a	Total:<br>37.8 (19–65)<br><br>Female: 10 (35.7%), Male: 18 (63.1%)<br><br>N/a	5 years	PION	Spontaneous Dissection<br><br>N/a	N/a	N/a	Angiography: 5<br><br>MR; Doppler ultrasonography and MRA: 23	N/a
Babovic et al., 2005	USA	Case Report	1<br><br>1 (100%)<br><br>N/a	43<br><br>Female<br><br>Right<br><br>(Bilateral CAD)	N/a	AION	Traumatic Dissection<br><br>N/a	Same day	3 months	MRI and MRA	Bilateral internal carotid arteries, with 50% stenosis on the right side
Koch et al., 2005	USA	Case Report	1<br><br>1 (100%)<br><br>N/a	53<br><br>Male<br><br>Right	N/a	PION	Spontaneous Dissection<br><br>N/a	24 hours	3 months	Angiography	N/a
Kawabe et al., 2009	Japan	Case Report	1<br><br>1 (100%)<br><br>N/a	73<br><br>Male<br><br>Left	N/a	PION	Spontanous Dissection<br><br>1 cm above the bifurcation	N/a	N/a	Carotid artery Doppler and Angiography	100%

First Author	Country	Study Design	Total Sample Size (n.)<br><br>Number of Patients with ION (n. and %)<br><br>Number of Patients of control groups (n. and %)	Age (years, mean ± SD or mean and range or mean age and 95% CI)<br><br>Gender (n. and %)<br><br>Involved Side	Study Time	Type of ION	Type of Dissection and occlusion site	Mean time between ION onset and first consultation	Follow-up Duration	Diagnostic investigation used for diagnosis	Stenosis severity
Lysandropoulos et al., 2010	Switzerland	Case Report	1<br><br>1 (100%)<br><br>N/a	27<br><br>Female<br><br>Right	N/a	PION	Spontanous Dissection<br><br>N/a	N/a	6 months	Carotid artery Doppler and CTA	Subocclusive dissection
Anders et al. 2014	USA	Case Report	1<br><br>1 (100%)<br><br>N/a	35<br><br>Male<br><br>Left	N/a	PION	Traumatic Dissection<br><br>Cavernous segment	Immediate	N/a	CTA	100%
Jah et al., 2019	India	Case Report	1<br><br>1 (100%)<br><br>N/a	55<br><br>Male<br><br>Right	N/a	AION	Spontanous Dissection<br><br>N/a	1 month	N/a	Doppler ultrasonography	100%
Zheng et al., 2020	China	Case-Report	1<br><br>1 (100%)<br><br>N/a	44<br><br>Female<br><br>Right	N/a	AION/ PION or a combination of thereof	Spontaneous Dissection<br><br>N/a	N/a	5 months	CTA and angiography	90%
Lains et al., 2021	USA	Case-report	1<br><br>1 (100%)<br><br>N/a	64<br><br>Male<br><br>Right	N/a	AION	Spontaneous Dissection<br><br>N/a	4 days	18 months	CTA	100%

Abbreviations: ION: Ischemic Optic Neuropathy; PION: Posterior Ischemic Optic Neuropathy; AION: Anterior Ischemic Optic Neuropathy; Wks.: Weeks, N: number; SD: Standard Deviation; ICA: Internal carotid artery; MRA: Magnetic resonance angiography; CTA: Computed tomography angiography, CI: Confidence interval; N/A: Not applicable; CAD: Carotid artery dissection
